# Cardiovascular Magnetic Resonance GUIDEd management of mild-moderate left ventricular systolic Heart Failure (CMR GUIDE HF): study protocol for a randomised controlled trial

**DOI:** 10.1186/1532-429X-17-S1-P191

**Published:** 2015-02-03

**Authors:** Joseph Selvanayagam, Sanjay K Prasad, Andrew D McGavigan, Graham Hillis, Werner Jung, Henry Krum

**Affiliations:** Flinders Medical Centre, Bedford Park, SA Australia; Flinders University, Adelaide, SA Australia; Sydney University, Sydney, NSW Australia; Royal Brompton Hospital, London, UK; Schwarzwald-Baar Klinikum, Villingen-Schwenningen, Germany; Monash University, Melbourne, VIC Australia

## Background

Current heart failure guidelines recommend insertion of implantable cardiac defibrillators (ICD) for primary prevention of sudden cardiac death (SCD) in patients whose left ventricular ejection fraction is (LVEF) ≤ 35% on maximally tolerated medical therapy. However, the majority of sudden cardiac death (SCD) in patients with heart failure occurs in those with mild-moderate systolic heart failure (LVEF 36%-50%) who currently do not qualify for an ICD. At present, our tools to reliably risk stratify patients with mild-moderate heart failure to predict the likelihood of SCD are limited. Recent data however suggest that ventricular scar and/or replacement fibrosis, reliably identified on Cardiac Magnetic Resonance Imaging (CMR), forms a substrate for malignant arrhythmia, thus potentially identifying a group at increased risk of SCD. The **primary aim** of the **C**ardiovascular **M**agnetic **R**esonance **GUIDE**d management of mild-moderate left ventricular systolic **H**eart **F**ailure (CMR GUIDE HF) trial is to test the hypothesis that among patients with mild-moderate heart failure, a routine CMR guided management strategy of implantable defibrillator (ICD) insertion is superior to a conservative strategy of standard care.

## Methods

A multi-centre randomized control trial based in Australia and Europe, enrolling 428 patients with mild to moderate left ventricular systolic dysfunction, on optimal heart failure therapy. Participants will be recruited through heart failure clinics and echocardiography databases to undergo CMR assessment for myocardial scar and fibrosis. The study will have two arms: Firstly, those with myocardial scar/fibrosis will be enrolled into a prospective, blocked, randomized control trial to receive either primary prophylaxis ICD therapy or an implantable loop recorder (ILR). Secondly, a further 521 patients without scar/fibrosis will be enrolled into an observational registry. Follow-up with be at 3, 6, 12, 24 and 36 months for the implant groups (ICD and ILR) and at 12, 24 and 36 months for the registry group.

## Results

The primary endpoint will be time to sudden cardiac death, or syncopal ventricular arrhythmia during an average 3-year follow-up. The secondary endpoints consist of sudden cardiac death, syncopal ventricular arrhythmia, all-cause mortality, quality of life assessed by Minnesota Living with Heart Failure Questionnaire, heart failure related hospitalizations and a cost utility analysis.

## Conclusions

This study will use **CMR as a novel tool for risk stratification** by directly imaging the scar which forms a critical substrate for ventricular arrhythmia. The results of this study will result in a **highly significant advance in knowledge in this field** by providing new information about the potential benefits of primary prevention ICD in mild-moderate LV systolic dysfunction. If proven, it would radically alter the way in which patients are selected for ICD therapy.

## Funding

Biotronik Australia.

NHMRC Govt of Australia.

**Figure 1:**
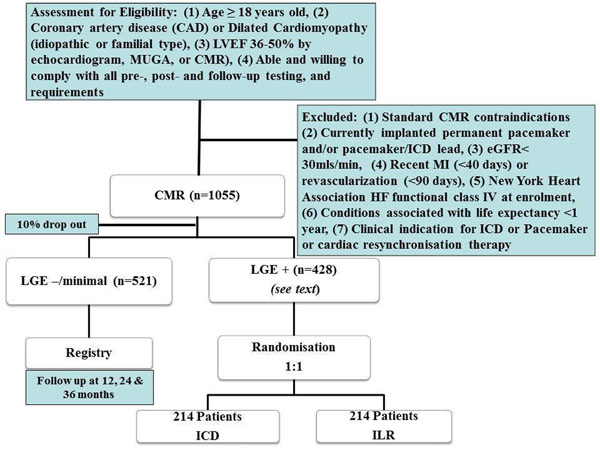
Figure 1

